# Draft genome of Mn(II)-oxidizing bacterium *Hydrogenophaga* sp. ZJX-1 isolated from coastal sediments in Liaoning, China

**DOI:** 10.1128/mra.01217-25

**Published:** 2025-12-22

**Authors:** Yu Liu, Fei Li, Jiaxi Zang, Hao Zhou

**Affiliations:** 1 Key Laboratory of Industrial Ecology and Environmental Engineering (Ministry of Education), School of Chemical Engineering, Ocean and Life Sciences, Panjin Campus, Dalian University of Technology, Panjin, China; Montana State University, Bozeman, Montana, USA

**Keywords:** manganese-oxidizing bacteria, genome sequencing, *Hydrogenophaga*, marine sediments

## Abstract

The draft genome sequence of *Hydrogenophaga* sp. ZJX-1, a Mn(II)-oxidizing bacterium isolated from coastal sediments of Liaodong Bay, China, consists of 5,014,722 bp with a G+C content of 65.94% and an N50 of 671,809 bp. Genome annotation suggests a potential coupling between Mn(II)-oxidation and carbon/nitrogen cycling.

## ANNOUNCEMENT

*Hydrogenophaga* species are gram-negative, flagellated, straight to slightly curved rods capable of growth in aerobic or facultatively anaerobic environments (1). Many members of this genus are chemoautotrophs that oxidize hydrogen as an energy source (2). Furthermore, the genus includes strains capable of aromatic hydrocarbon degradation and Mn(II) oxidation (3, 4). In this study, a Mn(II)-oxidizing bacterium (MnOB), *Hydrogenophaga* sp. ZJX-1, was isolated from surficial coastal sediments of Liaoning, China (122.11°E, 40.67°N). Approximately 10 g of sediment was suspended in 100 mL sterile saline, serially diluted (10⁻¹−10⁻⁶), spread onto modified minimal salt medium (mMSM) agar plates (with 20 g/L agar and 0.02 g/L MnCl_₂_·4H_₂_O), and incubated at 30℃ for 7 days (5). Brown colonies were restreaked on mMSM agar at least three times to obtain pure cultures, which were confirmed as MnOB using the Leucoberbelin Blue assay (6). Notably, *Hydrogenophaga* sp. ZJX-1 grew in mMSM without added organic carbon and in mMSM supplemented with 0.1% (w/v) glucose, indicating autotrophic and heterotrophic growth. It also grew in the absence of an external nitrogen source, suggesting a potential for nitrogen fixation.

Cells were harvested by centrifugation after 96 h of cultivation at 30°C in mMSM supplemented with 0.1% (w/v) glucose. Genomic DNA was extracted using the DNeasy PowerSoil Pro Kit (Qiagen) following the manufacturer’s instructions. The paired-end library was prepared with the Illumina Nextera DNA Library Prep Kit, and 2 × 150 bp reads were generated on an Illumina NovaSeq 6000 platform (Novogene, Tianjin, China). Raw reads (1,384 Mb) were filtered with readfq v10, removing reads with >40% low-quality bases (Q < 20), >10% ambiguous bases (N), and >15 bp adapter overlap; duplicate and putative host-derived reads were also discarded (7). In total, 1,111 Mb of clean data was retained, with Q20 and Q30 values of 97.67 and 93.49%, respectively. Preliminary assemblies were generated with SOAPdenovo2 v2.04, SPAdes v4.2.0, and ABySS v2.3.10 to improve contiguity by combining assemblies from multiple algorithms. These assemblies were merged using CISA, and remaining gaps were filled with GapCloser v1.2.1 (8–11). Fragments shorter than 500 bp were removed, and the final assembly was functionally annotated using the National Center for Biotechnology Information Prokaryotic Genome Annotation Pipeline (PGAP) v6.9 (12), which provided the gene counts and locus tags reported in this study. In addition, GeneMarkS v4.17 was used for gene prediction, and Kyoto Encyclopedia of Genes and Genomes was used for pathway assignment (13–15). Default parameters were used for all tools unless otherwise specified.

Based on EzBioCloud analysis, the 16S rRNA gene sequence of strain ZJX-1 showed the highest similarity (99.64%) to *H. aromaticivorans* D2P1 (4, 16). The final genome assembly consisted of 14 scaffolds with 222× sequencing coverage, a total size of 5,014,722 bp, a G + C content of 65.94%, and an N50 of 671,809 bp. Assembly quality assessed by CheckM v1.2.3 showed 99.39% completeness with 1.66% contamination (17). A total of 4,655 protein-coding genes, 52 tRNAs, and 12 rRNAs (four copies each of 23S, 16S, and 5S) were predicted. In addition, several proteins putatively associated with Mn(II) oxidation were identified by mmseqs2 through homologous alignment (18, 19). These sequences included stKatG, BoxA, CueO, MoxA, and three AHPs (Table 1). The genome also harbors the hydrogenase gene cluster *hypABCDEFG*, suggesting the potential to utilize hydrogen, along with a complete Calvin-Benson-Bassham cycle and nitrogen fixation pathway. These genomic features indicate that ZJX-1 may couple Mn(II)-oxidation with the carbon and nitrogen fixation processes.

**TABLE 1:** Candidate Mn(II)-oxidation protein in *Hydrogenophaga* sp. ZJX-1 and their relationships to experimentally characterized Mn-oxidizing proteins

Protein	RefSeq accessions (ZJX-1)	Locus tag (ZJX-1)	Organism	Protein accession	E-value (MMseqs2)	Reference
StKatG	WP_420050843.1	ACN5W5_RS23550	*Salinicola tamaricis* F02	WP_242495723.1	8.31E−246	(20)
BoxA	WP_420050286.1	ACN5W5_RS20440	*Arthrobacter* sp*.* QXT-31	ANG58850.1	8.16E−84	(21)
CueO	WP_420049694.1	ACN5W5_RS17090	*Escherichia coli* K-12	NP_414665.1	1.31E−55	(22)
MoxA	WP_420049672.1	ACN5W5_RS16980	*Pedomicrobium* sp. ACM 3067	CAJ19378.1	3.26E−197	(23)
AHP	WP_420049254.1	ACN5W5_RS14910	*Roseobacter* sp. AzwK-3b	AKH40935.1	5.80E−302	(24)
AHP	WP_420049253.1	ACN5W5_RS14905	*Roseobacter* sp. AzwK-3b	AKH40935.1	0	(24)
AHP	WP_420048182.1	ACN5W5_RS08875	*Pseudomonas putida* GB-1	ABY99245.1	0	(25)

## Data Availability

Raw genome sequencing data for *Hydrogenophaga* sp. ZJX-1 are deposited in the NCBI Sequence Read Archive (SRA) under accession number SRR36217927 as part of BioProject PRJNA1232854 and BioSample SAMN47251502. The draft genome assembly is available in GenBank under accession GCA_048590325.1 (WGS accession JBMBRA000000000.1). The 16S rRNA gene sequence has been deposited under accession PX622510.
